# Global Media Narratives of Shock and Perceptions of Electroconvulsive Therapy

**DOI:** 10.1192/j.eurpsy.2026.11450

**Published:** 2026-07-31

**Authors:** A. A. Rehman, B. R. Carr

**Affiliations:** University of Florida, Gainesville, FL, United States

## Abstract

**Introduction:**

Electroconvulsive therapy (ECT) remains one of the most effective interventions for severe and treatment-resistant depression. Yet its public image is shaped less by clinical efficacy than by the potent symbolic force of its media portrayals. Across national contexts, ECT is often represented not as healing but as coercive, punitive, or archaic. These visual and narrative constructions influence stigma, deter patients, and generate clinical ambivalence. Nevertheless, they remain underexplored in cross-cultural comparison.

**Objectives:**

To analyze representations of ECT in international film, television, digital media, and video games, with attention to the symbolic grammar and cultural anxieties that mediate its public perception. This review aims to expose how dramatized tropes displace therapeutic realities and reframe ethical discourse surrounding neuromodulation.

**Methods:**

A comparative literature and media analysis was conducted using indexed databases (PsycINFO, PubMed), drawing from scholarship from the years 1998 - 2024 on ECT representations in Bollywood cinema, Singaporean medical dramas, Australian independent films, American popular media, and video games. Qualitative thematic synthesis identified symbolic recurrences and sociocultural divergences, with interpretive emphasis on visual rhetoric, narrative context, and ethical subtext.

**Results:**

Four dominant motifs emerged:

- Punitive Spectacle – ECT appears as an instrument of state or institutional control, especially in institutionalized authoritarianism or carceral settings.
- Embodied Violation – Cinematic depictions emphasize convulsions, restraint, and bodily trauma, encoding ECT as an assault on personhood.
- Moral Disciplining – Recipients are framed as deviant or socially transgressive, positioning ECT as a form of behavioral correction.
- Absent Consent – Informed consent is routinely elided, with portrayals emphasizing helplessness, silence, and irreversibility.

**Conclusions:**

Media portrayals of ECT function as cultural pedagogy, reinforcing stigma through symbolic violence and ethical erasure. The remedy lies not only in patient education but also in cultural re-narration. Psychiatric institutions must engage critically with these public scripts. This includes acknowledging their historical roots and semiotic power, while actively asserting alternative narratives that emphasize autonomy, consent, and therapeutic dignity.

**Disclosure of Interest:**

None Declared

